# Do You Feel Included? A Latent Profile Analysis of Inclusion in the Chinese Context

**DOI:** 10.3389/fpsyg.2022.692323

**Published:** 2022-03-02

**Authors:** Jiaojiao Qu, Mengcheng Wang

**Affiliations:** ^1^School of Business Administration, Huaqiao University, Quanzhou, China; ^2^Department of Psychology, Guangzhou University, Guangzhou, China

**Keywords:** inclusion, latent profile analysis, diversity, the Chinese context, teachers

## Abstract

Although the importance of inclusion has been firmly supported by prior studies, the question of whether certain subgroups exist in the workplace whose members feel more or less included remains under-explored, limiting our understanding of how an inclusive workplace can be achieved. To address this gap, the current study conducted a latent profile analysis (LPA) to obtain evidence-based information regarding employees’ sense of inclusion in their organizations. Using a sample of 1,168 participants engaged in multiple industries in China, we identified three profiles of inclusion, with a largest proportion feeling a moderate level of inclusion (69.5%), a smaller proportion feeling a high level of inclusion (22.7%), and a tiny proportion feeling a low level of inclusion (7.8%). The three profiles differ significantly on key variables, demonstrating that the group feeling more included tends to be more aged and tenured, hold a higher educational degree, work in the high-tech sector, and come from a developed area. Such a group also shows more engagement in their work but less exhaustion, feels that they have more opportunities for development, and gains more support from colleagues and supervisors. Our findings point to the existence of subgroups of inclusion within the Chinese context and highlight the characteristics of these profiles, which in turn shed lights on how we can reach the goal of inclusion.

## Introduction

The workforce has been becoming increasingly culturally diverse (Moore et al., 2020). Although diversity management has been a topic of interest in theory and practice for more than 40 years (Chung et al., 2020), attention has shifted more recently in terminology and intent from “diversity” to “inclusion” (Mor Barak et al., 2016; Roberson et al., 2017; Shore et al., 2018; Ashikali et al., 2020; Chung et al., 2020), moving scholars and practitioners from focusing solely on the “what” and “who” to looking deeper into the “how” and “why” (Mor Barak et al., 2016; Shore et al., 2018; Rice et al., 2021).

Inclusion refers to “the degree to which an employee perceives that he or she is an esteemed member of the work group through experiencing treatment that satisfies his or her needs for belongingness and uniqueness” (Shore et al., 2011, p. 1265). The appeal of inclusion lies in its positive stance in terms of appreciating and valuing individual differences (Ferdman and Deane, 2014), enabling everyone to experience acceptance of their identities and ideas, to feel a part of the system in both formal and informal ways, and to sense that their voices and opinions are welcomed at every level of decision-making (Mor Barak et al., 2016; Chung et al., 2020).

Organizational practices, such as providing mechanisms for members to voice, participate, communicate, and develop (Offerman and Basford, 2014; Sabharwal, 2014; Tang et al., 2015), as well as supporting from their supervisors (Tang et al., 2015) and peers (Offerman and Basford, 2014), are thought to cultivate an individual’s perception of inclusion in the workplace. Additionally, demographic factors, such as gender, age, ethnicity, religious, education, and regional origin, are also found to be related to an individual’s perception of inclusion (Mor Barak et al., 2001; Cho and Mor Barak, 2008; Mousa et al., 2020). While inclusion has been regarded as the root of psychosocial wellbeing (Mor Barak and Cherin, 1998), comprising low exhaustion and high engagement (Bakker, 2014). It has been found to increase employee commitment (Hwang and Hopkins, 2015; Shore et al., 2018), job satisfaction (Brimhall et al., 2014; Hwang and Hopkins, 2015; Shore et al., 2018), organizational citizenship behavior (Ashikali and Groeneveld, 2015; Shore et al., 2018), creativity (Li et al., 2017; Chung et al., 2020), meaningfulness of work (Mousa et al., 2021), and job performance (Chung et al., 2020), while decrease interpersonal conflict (Nishii, 2013), turnover (Brimhall et al., 2014; Hwang and Hopkins, 2015; Shore et al., 2018), and discrimination (Dwertmann and Boehm, 2016).

Although the importance of inclusion has been consistently supported by empirical research (e.g., Nishii, 2013; Dwertmann and Boehm, 2016; Chung et al., 2020) and meta-analyses (Mor Barak et al., 2016; Holmes et al., 2021), the way to foster this sense of inclusion and cultivate an inclusive workplace remains under-examined (Shore et al., 2018; Ashikali et al., 2020; Rice et al., 2021). Research has thus begun to probe into the factors that may contribute to one’s sense of inclusion. In particular, the evidence-based findings regarding which subgroups feel more or less included in an organization are quite insightful and can guide us on where our efforts should be invested for the purpose of achieving the goal of inclusion (Brimhall and Saastamoinen, 2020).

Latent profile or class analysis (LPA/LCA) is a person-centered classification analytical tool used to identify homogeneous latent classes or subgroups within a large heterogeneous sample (Hagenaars and McCutcheon, 2002; Gabriel et al., 2015). In contrast to a variable-centered approach, LPA/LCA allows us to detect profiles that differ qualitatively or quantitatively in combinations of variables (Wang and Hanges, 2011; Woo et al., 2018), and identify how the different subgroups are differentially linked to antecedents and outcomes (Wang and Hanges, 2011). This is a useful approach in identifying meaningful subtypes that are more homogeneous smaller groups within the larger heterogeneous group (da Silva et al., 2019; Vaziri et al., 2020; Wang et al., 2020).

Based on the Inclusion–Exclusion Scale (Mor Barak, 2016), Brimhall and Saastamoinen (2020) explored the topologies of inclusion with LPA using a sample from the public departments in the United States and identified two subgroups (i.e., those who feel less valued vs. those who feel more valued). Although these findings are meaningful and provocative, however, the inclusion typologies are needed to be investigated further as the number and meaning of profiles depend greatly upon sample characteristics and assessment tools (Jason and Glenwick, 2016; Wang et al., 2020). The tool used in their study was the 15-item scale developed by Mor Barak (2016), which measures the decision-making process, information networks, and one’s level of participation, and is more indicative of the causes of inclusion rather than evaluating one’s sense of inclusion *per se*. Meanwhile, the sample was small with only 213 participants, and from a single source focusing only on public organizations. The lack of representative sample in their study limited the validation of the profiles.

To address the limitations of the extant research, the current study aimed to use LPA to identify the inclusion profiles using the Work Group Inclusion Scale (Chung et al., 2020) and drawing on a larger sample with more participants coming from diverse organizations and multiple professions in a non-Western context. The 10-item Work Group Inclusion Scale, with five items measuring belongingness and five items measuring uniqueness, perfectly aligns with the established definition of inclusion comprising belongingness and uniqueness (Shore et al., 2011). This measure is expected to demonstrate the profiles of inclusion better. Furthermore, a representative sample is crucial to the validation of the profiles obtained. An LPA using larger sample with participants coming from various organizations and engaging in different professions is more likely to generate a more robust classification. Importantly, the Chinese culture is characterized by high collectivism (Hofstede, 1980), conformity (Goncalo and Staw, 2006), and harmony orientation derived from the dominant Confucian values (Leung et al., 2002; Kang et al., 2017; Horak and Yang, 2019). It is not thought to be as diverse as the Western contexts (Cho and Mor Barak, 2008). In this regard, conducing an LPA in such a situation can potentially extend the previous studies grounded in the Western context and identify unique profiles pertinent to the Chinese culture.

Therefore, our pilot study contributes to the existing research by identifying the patterns of inclusion in a non-Western context with a more representative sample and providing a more in-depth understanding of inclusion profiles by using a more valid measure of inclusion, subsequently shedding new light on how we can establish an inclusive workplace.

## Materials and Methods

### Participants and Procedure

Data for this study were collected from August 25 to September 10 in 2021. Participants were recruited from eight large-scale companies (more than 20,000 employees) in southeast China. Of these companies, three are in the manufacturing industry, three in the service industry, and two in the high-tech industry. After obtaining supports from the HR managers in each organization, a link to our online questionnaires on an online survey platform in China^1^ was sent to the employees’ WeChat group for each organization.^2^ Anyone who was willing to participate in survey was able to answer the questionnaires on their smart phone through this link. Participants were told their involvement was voluntary and anonymous. Each one who completed the questionnaires would receive a reward of ¥5 yuan.^3^ Questionnaires submitted too quickly (i.e., completed in less than 180 s) or too slowly (i.e., took more than 45 min to complete) were considered to have been answered carelessly and were thus deleted.

We received 1,251 responses in total and deleted 83 questionnaires due to a large number of missing answers or the same answers. Our final sample was comprised of 1,168 participants. Of them, 53.6% were male, the most majority had achieved an undergraduate degree or higher (85.1%), their job tenure was from 3 months to 26 years (*M* = 3.09, *SD* = 2.33), and all participants were between 20 and 50 years of age (*M* = 26.8, *SD* = 4.71). In terms of their sector, 303 participants (25.9%) engaged in the manufacturing industry, 422 participants (36.1%) were in the service industry, and 443 participants (37.9%) worked in the high-tech industry. As for their regional origins, 408 participants (34.9%) were from developed areas, 264 participants (22.6%) came from moderately developed areas, and the remaining 496 participants (42.5%) grew up in the less developed areas. The demographic information is presented in Table 1.

**Table 1:** Descriptive statistics of demographic variables.

Variables		*n*	%
Gender	Female	542	46.4
	Male	626	53.6
Age	20–25 years	559	47.9
	26–30 years	384	32.8
	31–35 years	161	13.8
	36–40 years	58	4.9
	41–45 years	4	0.3
	46–50 years	2	0.2
Tenure	Less than 1 year	612	52.4
	1–3 years	162	13.9
	4–6 years	127	10.9
	More than 6 years	267	22.9
Education	Below undergraduate degree	174	14.9
	Undergraduate degree	294	25.2
	Master’s degree	223	19.1
	Above master’s degree	477	40.8
Industry	Manufacturing	303	25.9
	Service	422	36.1
	High-tech	443	37.9
Region	Developed area	408	34.9
	Moderately area	264	22.6
	Less developed area	496	42.5

### Measures

All the scales used in the current study were originally developed in English and underwent a back translation process (Brislin, 1986). Items were first translated into Chinese by one of the authors of this study. Then, a bilingual PhD student majoring in Human Resource Management (HRM) translated them back into English independently. After that, another bilingual scholar in the HRM field compared the Chinese and English versions of all scales and made any necessary modifications to resolve any minor discrepancies by discussing them with both the previous two translators.

#### Inclusion

To assess the individual’s perception of inclusion, a 10-item scale developed by Chung et al. (2020) was used. It includes two dimensions, with a sample item being, “I am treated as a valued member of my work group” for *belongingness* (*α* = 0.951), and “I can share a perspective on work issues that is different from my group members” for *uniqueness* (*α* = 0.967). All items were rated on a scale ranging from 1 (strongly disagree) to 6 (strongly agree). Cronbach’s alpha for the total scale was 0.976. CFA results showed that the measure fit our data well (*χ*^2^ = 359.814, *df* = 34, CFI = 0.945, TLI = 0.928, SMR = 0.031).

#### Exhaustion

A five-item subscale adopted from the Job Demands-Resources Questionnaire (JD-RQ; Bakker, 2014) was used to measure individuals’ level of exhaustion. A sample item is, “There are days when I feel tired before I arrive at work” (1 = “Never” and 7 = “Always”). Cronbach’s alpha for this scale was 0.955. The CFA showed satisfactory fit (*χ*^2^ = 209.50, *df* = 5, CFI = 0.951, TLI = 0.902, SMR = 0.033).

#### Engagement

We used the nine-item subscale, also adopted from the JD-RQ (Bakker, 2014) to assess individuals’ level of engagement. A sample item is, “I am immersed in my work” (1 = “Never” 7 = “Always”). The same Cronbach’s alpha for this measure was 0.905. CFA results indicated that it fit the data well (*χ*^2^ = 308.261, *df* = 27, CFI = 0.954, TLI = 0.932, SRMR = 0.064).

#### Opportunities for Development

The subscale with three items also adopted from the JD-RQ (Bakker, 2014) was used to measure individuals’ level of opportunities for development. A sample item is, “In my work, I have the opportunity to develop my strong points” (1 = “Strongly disagree” and 5 = “Strongly agree”). Cronbach’s alpha for this measure was 0.906. CFA results indicated that it fit the data perfectly (*χ*^2^ = 0, *df* = 0, CFI = 1, TLI = 1, SRMR = 0).

#### Support From Colleagues

We used three-item subscale adopted from the JD-RQ (Bakker, 2014) to measure individuals’ perceived support from supervisors. A sample item is, “If necessary, can you ask your colleague for help?” (1 = “Never” and 6 = “Very often”). Cronbach’s alpha for this instrument was 0.924. CFA results indicated that it fit the data perfectly (*χ*^2^ = 0, *df* = 0, CFI = 1, TLI = 1, SRMR = 0).

#### Support From Supervisor

To assess individuals’ perceived support supervisor, we used the five-item coaching subscale also adopted from the JD-RQ (Bakker, 2014). A sample item is, “My supervisor uses his/her influence to help me solve problems at work” (1 = “Strongly disagree” and 5 = “Strongly agree”). Cronbach’s alpha for this instrument was 0.988. CFA results indicated that it fit the data well (*χ*^2^ = 57.388, *df* = 5, CFI = 0.985, TLI = 0.970, SRMR = 0.022).

### Statistical Analysis

Descriptive statistical and internal consistency analyses were conducted using SPSS v.21 (IBM SPSS, 2012). CFA and LPA were conducted using Mplus 7. (Muthén and Muthén, 2017).

To examine the structural validity of the constructs involved, we conducted a series of CFA using the robust maximum likelihood estimator. Several fit indices were considered: Comparative Fit Index (CFI), Tucker–Lewis index (TLI), and Standardized Root Mean Square Residual (SRMR).

To identify homogeneous groups (latent profiles), LPA was performed on participates’ scores on each item of inclusion. A mixture modeling approach was used as there were both categorical and continuous variables of interest (Neely-Barnes, 2010). A series of LPA models (from one-class to five-class) were estimated using robust maximum likelihood (MLR), beginning with a one-class model, and iteratively adding the number of latent profiles until there was no further improvement in model fit. To avoid Local Likelihood Maxima, 200 random sets of starting values were set initially, together with 50 final stage optimizations (Muthén and Muthén, 2017; Wang et al., 2020).

To determine the optimal model, we compared several fit indices across models with different profiles, including the Akaike Information Criterion (AIC; Akaike, 1987), the Bayesian Information Criterion (BIC; Schwarz, 1978), and the Sample Size-Adjusted Bayesian Information Criterion (SSA-BIC; Sclove, 1987). With these three indices, a lower model value is indicative of a better fit. We also used Entropy to assess the quality of the classifications, where a value higher than 0.80 suggests that the accuracy of the classification was over 90% (Lubke and Muthén, 2007). We also performed the Lo–Mendell–Rubin Test (LMR; Lo et al., 2001) and the Bootstrap Likelihood Ratio Test (BLRT; McLachlan and Peel, 2004), with significant LMR and BLRT results (*p* < 0.05) indicating that the specified k-profile solution was significantly better than the k-1 profile solution (Nylund et al., 2007). In addition, the average probabilities of class membership were also considered. If the derived model with the average probabilities of all classes was higher than 0.80, this was considered to be an acceptable fit (Vermunt and Magidson, 2002). As these indices are sometimes contradictory, we also depicted elbow plots of BIC to examine where the curve flattened to determine the optimal classifications. Finally, as identified patterns and profiles should be interpretable in theory (Nylund et al., 2007; Marsh et al., 2009; Neely-Barnes, 2010), we examined the substantive meanings for each class once the optimal model was identified, and then named them correspondingly to offer an abstract description of each class.

To detect the differentiations among the profiles of the determined optimal model, we further tested the pattens in terms of their distinct associations with the relevant variables. Using Mplus 7.4, the class differences on key variables (i.e., gender, age, tenure, education, industry, region, exhaustion, engagement, opportunity for development, support from colleague, and support from supervisor) across subgroups were examined through the modified Bolck-Croom-Hagenaars (BCH; Bolck et al., 2004) and categorical distal outcome (DCAT; Lanza et al., 2013; Asparouhov and Muthén, 2014) methods. The former allows for the analysis of continuous variables and accounts for unequal variances among the variables and measurement errors of the latent classes (Craig and Moretti, 2019; Wang et al., 2020), while the latter is used to investigate the differences for categorical variables across latent profiles (Asparouhov and Muthén, 2014).

## Results

The CFA results (see Table 2) show that the expected six-factor model involving inclusion, exhaustion, engagement, opportunity for development, support from colleagues, and support from supervisor, fitted the data best (*χ*^2^ = 7356.722, df = 545, CFI = 0.927, TLI = 0.912, SMR = 0.008), supporting the discriminant validity of our measures.

**Table 2:** Results of confirmatory factor analyses.

Model	*χ* ^2^	*df*	CFI	TLI	SRMR
Full factors					
INC, ENG, EXH, OFD, SFS, SFC	7356.722	54	0.927	0.912	0.08
Five factors					
INC, ENG, EXH, OFD + SFS, SFC	8915.552	550	0.763	0.744	0.12
Four factors					
INC, ENG, EXH + OFD + SFS, SFC	11291.831	554	0.689	0.666	0.18
Three factors					
INC, ENG + EXH + OFD + SFS, SFC	14292.904	557	0.584	0.556	0.13
Two factors					
INC, ENG + EXH + OFD + SFS + SFC	16105.348	559	0.531	0.501	0.13
Single factor					
INC + ENG + EXH + OFD + SFS + SFC	19728.260	560	0.442	0.407	0.17

*INC, Inclusion; EXH, Exhaust; ENG, Engagement; OFD, Opportunity for development; SFC, Support from colleague; SFS, Support from supervisor; “+”: factor combination*.

Table 3 provides descriptive information for study variables, as well as the Pearson correlations between them.

**Table 3:** Correlation, means (*M*), and standard deviations (*SD*) for all variables (*N* = 1,168).

Variable	1	2	3	4	5	6	7	8	9	10	11	12
1. Gender^a^	1											
2. Age	−0.214^**^	1										
3. Tenure	0.037	0.696^**^	1									
4. Education^b^	−0.212^**^	0.545^**^	0.362^**^	1								
5. Industry^c^	−0.067^*^	0.442^**^	0.355^**^	0.429^**^	1							
6. Region^d^	−0.150^**^	−0.002	−0.105^**^	−0.205^**^	−0.118^**^	1						
7. Exhaustion	−0.148^**^	−0.154^**^	−0.167^**^	−0.271^**^	−0.220^**^	0.305^**^	1					
8. Engagement	−0.054	0.368^**^	0.314^**^	0.335^**^	0.416^**^	−0.067^*^	−0.353^**^	1				
9. Support from colleagues	−0.315^**^	0.458^**^	0.230^**^	0.583^**^	0.378^**^	−0.135^**^	−0.257^**^	0.291^**^	1			
10. Support from supervisor	0.119^**^	0.401^**^	0.349^**^	0.343^**^	0.459^**^	−0.088^**^	−0.296^**^	0.391^**^	0.494^**^	1		
11. Opportunity	−0.128^**^	0.385^**^	0.289^**^	0.417^**^	0.411^**^	−0.095^**^	−0.260^**^	0.413^**^	0.391^**^	0.317^**^	1	
12. Inclusion	0.008	0.453^**^	0.338^**^	0.408^**^	0.590^**^	−0.091^**^	−0.394^**^	0.446^**^	0.436^**^	0.542^**^	0.452^**^	1
Mean	1.54	26.77	3.09	3.46	2.12	2.20	3.11	3.91	3.57	2.87	4.63	4.12
*SD*	0.499	4.708	2.326	1.570	0.790	0.782	1.289	1.1165	1.322	0.926	0.594	0.926

a *1 = female, 2 = male*.

b *1 = below undergraduate degree, 2 = undergraduate degree, 3 = master’s degree, 4 = above master’s degree*.

c *1 = manufacturing, 2 = service, 3 = high-tech*.

d *1 = developed areas, 2 = moderately developed areas, 3 = less developed areas.*

* *p < 0.05 and*

** *p < 0.01*.

Table 4 presents the LPA model fit indices for the one- to five-profile models in the sample (*N* = 1,168). However, AIC, BIC, SSA-BIC, Entropy values, and LMR and BLRT results of each model suggest a good fit, thus we cannot determine an appropriate classification. Following the suggestions of Vaziri et al. (2020), we then graphed the elbow plots of the BIC of the five models to identify the ideal profile solution.

**Table 4:** Model fit indices of the latent profile analysis (*N* = 1,168).

Model	Log likelihood	Number of free parameters	AIC	BIC	SSA-BIC	Entropy	LMR*p*	BLRT*p*	Average latent class probabilities	Class proportions
1class	−16786.65	20	33636.31	33714.57	33651.04	_	_	_		
2class	−13444.88	31	26951.72	27108.72	27010.25	0.994	<0.001	<0.001	0.997–0.999	0.091/0.909
3class	−10121.30	42	20326.60	20539.25	20405.84	0.993	<0.001	<0.001	0.994–0.999	0.078/0.695/0.227
4class	−9280.36	53	18666.72	18935.05	18766.71	0.986	<0.01	<0.001	0.981–0.999	0.079/0.065/0.217/0.649
5class	−8685.19	64	17498.38	17822.41	17619.12	0.989	<0.01	<0.001	0.982–0.999	0.076/0.059/0.592/0.206/0.066

*AIC, Akaike information criteria; BIC, Bayesian information criteria; SSA-BIC, Sample size-adjusted BIC; LMRp, p value of the Lo–Mendell–Rubin Adjusted test; BLRTp, p value of the Bootstrap Likelihood Ratio Test*.

**Figure 1:**
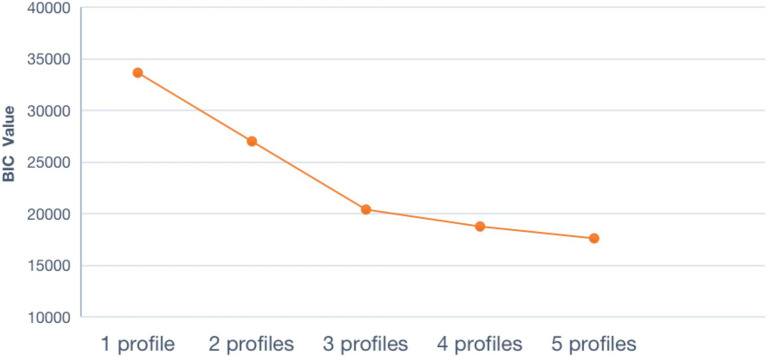
Elbow plot for Bayesian Information Criterion (BIC).

As can be seen in Figure 1, the elbow plot of the BIC shows that the slope of the curve flattens around the three-class point. The average probabilities of class membership for this classification were higher than 0.90 (ranging from 0.994 to 0.999, as seen in Table 4). We thus retained the three-class model as the optimal one.

**Figure 2:**
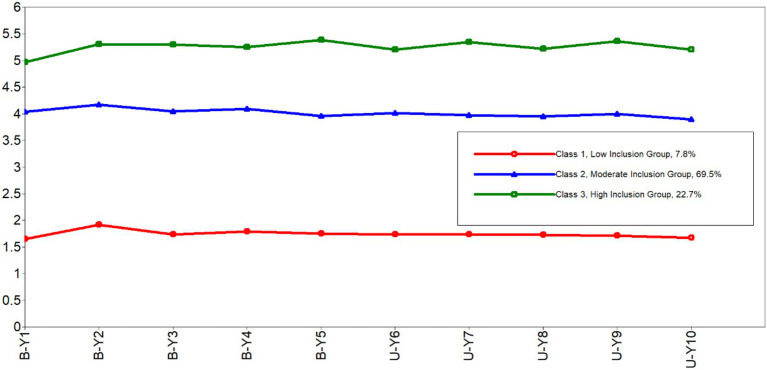
Latent profiles of inclusion.

Figure 2 depicts a graphic description of the three-class solution in our sample. Means and Standard Errors of the inclusion measures for each class are reported in Table 5. The majority of our sample (69.5%) belonged to a profile with moderate scores on all items of inclusion, and we therefore named the class the *moderate inclusion group* (Class 2). Class 3 (22.7%) was characterized by relatively high scores on each item, was thus named the *high inclusion group*. Although the remaining class (Class 1) accounted for only a small proportion of our sample (7.8%), it was a meaningful class with scores on all items being the lowest, thus was correspondingly named the *low inclusion group*.

**Table 5:** Comparisons of the three latent profiles on the outcome variables with DCAT and BCH (*N* = 1,168).

Variable		Class 1 (*n* = 91)	Class 2 (*n* = 812)	Class 3 (*n* = 265)	Class 1 vs. Class 2	Class 1 vs. Class 3	Class 2 vs. Class 3	DCATχ^2^	BCHχ^2^
Categorical	**Gender**				0.459	0.096	0.256	0.622	
	Female	0.494(0.053)	0.457(0.018)	0.475(0.032)					
	Male	0.506(0.053)	0.543(0.018)	0.525(0.032)					
	**Education**				498.87^***^	542.62^***^	62.09^***^	843.46^***^	
	Below undergraduate degree	0.506(0.052)	0.123(0.012)	0.106(0.019)					
	Undergraduate degree	0.450(0.052)	0.281(0.016)	0.095(0.019)					
	Master’s degree	0.000(0.000)	0.198(0.014)	0.234(0.026)					
	Above master’s degree	0.044(0.021)	0.398(0.017)	0.565(0.031)					
	**Industry**				880.29^***^	284.13^***^	2380.46^***^	2572.30^***^	
	Manufacturing	0.769(0.044)	0.294(0.016)	0.015(0.007)					
	Service	0.000(0.000)	0.524(0.018)	0.000(0.000)					
	High-tech	0.231(0.044)	0.191(0.015)	0.985(0.007)					
	**Region**				21.96^***^	21.36^***^	23.84^***^	44.02^***^	
	Developed area	0.209(0.043)	0.334(0.017)	0.446(0.031)					
	Moderately developed area	0.143(0.037)	0.264(0.016)	0.137(0.022)					
	Less developed area	0.648(0.050)	0.402(0.017)	0.417(0.030)					
Continuous	Age	21.582(0.190)	26.361(0.141)	29.801(0.328)	406.07^***^	470.41^***^	92.44^***^		627.44^***^
	Tenure	1.351(0.077)	2.913(0.076)	4.215(0.163)	209.25^***^	252.03^***^	51.97^***^		356.16^***^
	Exhaustion	4.576(0.115)	3.126(0.041)	2.558(0.079)	139.79^***^	208.39^***^	40.48^***^		209.35^***^
	Engagement	2.910(0.100)	3.797(0.031)	4.597(0.085)	71.42^***^	165.58^***^	78.08^***^		167.32^***^
	Opportunity for development	3.971(0.056)	4.615(0.019)	4.922(0.036)	118.73^***^	204.98^***^	57.19^***^		205.70^***^
	Support from colleague	1.959(0.115)	3.578(0.046)	4.105(0.056)	170.47^***^	281.89^***^	52.54^***^		284.80^***^
	Support from supervisor	1.965(0.094)	2.727(0.029)	3.633(0.045)	60.58^***^	257.06^***^	282.01^***^		393.73^***^

*Class 1 = Low inclusion group; Class 2 = Moderate inclusion group; Class 3 = High inclusion group*.

*** *p < 0.001*.

*Relations of the three latent profiles to categorical outcomes variable are presented as probability and standard error (SE); Relations of the three latent profiles to continuous outcomes variables are presented as M (SE). BCH*, *modified Bolck-Croom-Hagenaars method; DCAT*, *Categorical distal outcome method*.

Table 5 summarizes the DCAT and modified BCH results of the overall chi-square tests and chi-square statistics for pairwise comparisons between classes (*N* = 1,168) for the categorical and continuous outcome variables. As shown in Table 5, all comparisons on the external variables involved across the three subgroups were significant, with the exception of gender. Specifically, those in the high inclusion group (Class 3) tended to be more aged (*M* = 29.801, *SE* = 0.328) and tenured (*M* = 4.215, *SE* = 0.163), have obtained a master’s degree (23.4%, *SE* = 0.026) or above (56.5%, *SE* = 0.031), work in the high-tech industry (98.5%, *SE* = 0.007), and come from developed regions (44.6%, *SE* = 0.031). They also had the highest levels of engagement (*M* = 4.597, *SE* = 0.085), perceived opportunity for development (*M* = 4.922, *SE* = 0.036), support from their colleagues (*M* = 4.105, *SE* = 0.056) and supervisor (*M* = 3.633, *SD* = 0.045) while the lowest levels of exhaustion (*M* = 2.558, *SD* = 0.079). Those in the low inclusion group (Class 1) demonstrated almost the opposite patten. The profile of this group tended to be that they were relatively young (*M* = 21.582, *SE* = 0.190) and junior (*M* = 1.351, *SE* = 0.077), hold an undergraduate degree (45.0%, *SE* = 0.052) or below (50.6%, *SE* = 0.052), work in the manufacturing industry (76.9%, *SE* = 0.044), come from the less developed areas (64.8%, *SE* = 0.050), They had the lowest levels of engagement (*M* = 2.910, *SE* = 0.100), least likely to perceive the opportunity for development (*M* = 3.971, *SE* = 0.056), support from colleagues (*M* = 1.959, *SE* = 0.115) and supervisor (*M* = 1.965, *SE* = 0.094) while most likely to be exhaustion (*M* = 4.576, *SE* = 0.115). Finally, those in the moderate inclusion group (Class 2) tended to work in the service industry (52.4%, *SE* = 0.018) and come from moderately developed areas (26.4%, *SE* = 0.016). They exhibited a moderate level on all other foci variables.

Overall, the three-class profiles differ significantly on all distal outcome variables excluding gender. The pattens of the three classes regarding the links to these relevant variables demonstrate that a greater sense of inclusion in a group relates to more beneficial outcomes while fewer harmful consequences, suggesting the homogeneity within the classes and heterogeneity across classes.

## Discussion

To obtain evidence-based information regarding employees’ sense of inclusion, an LPA based on the scores of the Work Group Inclusion Scale (Chung et al., 2020) was conducted in a diverse sample of 1,168 Chinese employees. Three latent profiles were identified: the moderate inclusion group (Class 2) comprised the majority (69.5%) of the total sample, with moderate scores on all items of inclusion; the high inclusion group (Class 3) accounted for a smaller proportion of the sample (22.7%), scoring highest on all items; while the low inclusion group (Class 1) were only a tiny percentage of the sample (7.8%), scoring lowest on all items. The three subgroups were found to differ on the key variables, revealing more details regarding the patterns of inclusion experienced by Chinese employees.

Interestingly, our results showed that members of the high inclusion group tended to be highly educated with a master’s degree or above, work in the high-tech industry, and come from developed areas; members of the moderate inclusion group tended to work in the service industry, and come from moderately developed areas; while members of the low inclusion group tended to have an educational level of an undergraduate degree or below, work in the manufacturing industry, and come from less developed areas. These differentiations pertinent to educational attainment, industry, and regional origins across the three profiles could be due to the inequality resulting from the household registration system in China (the Hukou system). As a dominant means of managing population mobility, the Hukou system has caused the “rural–urban” divisions, determining one’s eligibility for certain state-provided services and welfare programs pertaining to education and employment, among others (Liu, 2005; Chan, 2010; Afridi et al., 2015). Those who are well educated are then more likely to get better jobs in prosperous industries. In such an institutional arrangement, members from less developed areas usually hold a rural status with fewer educational and employment opportunities, thus leading to a lower level of perceived inclusion in the workplace. Compared to them, members from moderately or highly developed areas are more likely to hold an urban status with better education opportunities and more decent jobs, thus leading them to perceiving a relatively higher level of inclusion in the workplace.

Counterintuitively, the current study did not find any differences on gender across the three profiles, contrary to the results of Brimhall and Saastamoinen (2020). Their findings revealed that the group felt less valued is more likely to be female than to be male. However, there were no such differences found across the three profiles in the current study. This could be attributed to the Women’s Liberation Movement in China began in the early 20th century (Zuo, 2013) and has had ongoing supported by the Chinese Communist Party. As a result, gender inequality has been to some extent eliminated (Zuo, 2013) with women having more equal rights and opportunities to education, political participation, and employment than before (Lee, 1995). Those women who participated in the current study all had access to decent job opportunities—some had even been hired by high-tech companies that require high levels of competence and talent. These women may thus have a sense of fair, equal, accepted, and recognized as male employees do in the workplace. Therefore, the three subgroups did not differ on gender.

Noticeably, the patterns of inclusion identified in the current research were characterized by the majority of participants feeling a moderate level of inclusion or below (Class 2 and Class 1, 77.3%), and only a smaller group feeling a high level of inclusion (Class 3, 22.7%). This could be ascribed to the seniority-based system embedded in Confucian ideals in China (Kang et al., 2017; Horak and Yang, 2019). In this type of social setting, juniors (inferiors) are expected to follow, obey, and respect their seniors (superiors; Cho and Mor Barak, 2008), and these seniors are thus more likely to feel higher inclusion in their organizations. In our study, the most majority of participants were juniors with younger ages (*M* = 26.77, *SD* = 3.09) and shorter job tenures (*M* = 4.71, *SD* = 2.33). As such, it could be that they currently are less likely to feel as respected and valued as their seniors, experiencing fewer wage increases and/or less career growth as their older, more tenured colleagues (Horak and Yang, 2019). Accordingly, they may not feel a higher sense of inclusion that comes through seniority, even though the majority do already experience a moderate sense of being included in their organizations. Similarly, those perceiving a high degree of inclusion (i.e., Class 3) tended to be relatively older and hold longer tenure in our study.

As expected, we verified that there were substantial and regular differences on distal outcome variables across the three profiles by showing that the group with a higher level of inclusion engaged more in work, perceived more opportunity for development, support from colleagues, and support from supervisor, while also experiencing less exhaustion. This result is consistent with previous studies that have suggested that a greater sense of inclusion is associated with more positive outcomes (Nishii, 2013; Hwang and Hopkins, 2015; Dwertmann and Boehm, 2016; Shore et al., 2018) and less negative outcomes (Nishii, 2013; Brimhall et al., 2014; Dwertmann and Boehm, 2016). The findings of the current study therefore support the significance of inclusion in the workplace.

### Implications

This study has several theoretical and managerial implications. First, the current study extended existing research by replicating the use of LPA to investigate profiles of inclusion outside a Western cultural context, and revealing the unique patterns in the Chinese cultural context. Unlike previous findings, where the identified subgroups comprised simply those who felt less vs. more included (Brimhall and Saastamoinen, 2020), our study found three subgroups, differentiated by ones experiencing a high, moderate, or low level of inclusion. Furthermore, while more than half of the sample belonged to the high inclusion subgroup in previous study (Brimhall and Saastamoinen, 2020), the majority in our study were found to be positioned in the moderate inclusion subgroup (69.5%), with a much smaller proportion (22.7%) were categorized into the high inclusion subgroup, and the low inclusion subgroup accounting for only a tiny proportion of the sample (7.8%). Meanwhile, the differences in demographic variables across the profiles reported in the current study were not the same as in the previous study (Brimhall and Saastamoinen, 2020). Although there were significant differences in sample age, tenure, and education in both studies, the profiles of inclusion differed in gender and race variables in the American context, while in the Chinese context they differed in industry and region variables, which could be a result of China’s unique Hukou system. This result points to preliminarily evidence of the cultural sensitivity of inclusion (Tang et al., 2017; Shore et al., 2018; Brimhall and Saastamoinen, 2020).

Second, our results different from those of the previous study imply that the measures used to assess inclusion may matter. For our study, we adopted the 10-item scale developed by Chung et al. (2020), which fits well with the academic definition of inclusion that includes belongingness and uniqueness, while Brimhall and Saastamoinen (2020) applied the 15-item scale developed by Mor Barak (2016) that focuses on the decision-making process, information networks, and one’s level of participation. Discrepancies in the findings of the two studies may be a result of different measures with different focuses when examining the patterns of inclusion.

Third, our results corroborate the extant findings that inclusion could foster the positive functions while limiting negative impacts in an organizational setting (Nishii, 2013; Mor Barak et al., 2016) by revealing that a higher sense of inclusion correlates with an increased level of engagement, as well as perceiving more opportunity for development, more support from colleagues and supervisors, but less exhaustion. This highlights the importance and necessity of creating an inclusive workplace.

Finally, as the measure used in the current study (Chung et al., 2020) comprises belongingness and uniqueness, our results offer useful insights for managers and practitioners into how an inclusive environment can be achieved. For example, taking the initiative to satisfy employees’ dual needs of feeling accepted (belongingness) and valued (uniqueness), signifying to all—particularly to those feeling less included (i.e., younger and more junior, less educated, working in the manufacturing industry, and coming from less developed areas)—that they are respected, appreciated, valued, and are encouraged to voice, participate in decision-making, and contribute to their organizations, are feasible ways to reach the goal of inclusion.

### Limitations and Future Directions

We must also acknowledge some limitations in our study. First, our sample only comprised only 1,168 employees, and it is unknown whether the same profiles could be generalized to other employees in different organizations, sectors, or regions. It is worth noting that the majority of the respondents in our study were younger than 40 years old. This may be attributed to the fact that the older employees are less embracing the digital tools than their younger peers, and thus more reluctant to participate in our survey through Wechat. Future research should seek to broaden the representativeness of the sample to cover more different age groups, professions, or regions to achieve more robust conclusions.

Second, we considered only some common demographic variables (i.e., age, gender, job tenure, education, industry, and region), but overlooked other prominent or salient demographic factors which could be pertinent to the perceptions of inclusion in the Chinese context, such as ethnicity, Hukou, and dialect (Tang et al., 2015). Future research should incorporate these demographic factors when performing an LPA to further unpack the characteristics of the profiles of inclusion and provide a more nuanced understanding of their composition.

Likewise, we focused on a limited numbers of external variables (i.e., exhaustion, engagement, opportunity for development, support from colleagues, and support from supervisor), which is not adequate to completely reveal the full attributes of inclusion. Future research could extend our research by incorporating more key external variables, such as safety, empowerment, identity, creativity, and performance, as well as the emergent crisis (e.g., COVID-19 pandemic) to illuminate the effects of inclusion profiles in more detail.

Finally, as our study was conducted within the Chinese context, although we identified the unique profiles relating to this specific culture, we cannot generalize our findings to a broader non-Western context. Future research could perform comparative studies across cultures and societies so as to provide a more comprehensive picture of the inclusion profiles in a non-Western context.

## Conclusion

The current study conducted an LPA on inclusion in the Chinese context as an initial exploration into the profiles of inclusion in a non-Western context. Using the 10-item Work Group Scale that fits well with the definition of inclusion comprising belongingness and uniqueness, our results identified three profiles of inclusion: a high inclusion group (22.7%), a moderate inclusion group (69.5%), and a low inclusion group (7.8%). Our findings suggest the uniqueness of these inclusion profiles pertaining to the Chinese context and confirm the importance of inclusion by revealing substantial differences across the subgroups on key variables. Through the use of the Work Group Inclusion Scale, this study also offers insights into how we can cultivate an inclusive workplace and ensure all member are included, that is, by striving to fulfill individuals’ needs for belongingness and uniqueness. Future research could advance our knowledge of the inclusion typologies by enhancing the representativeness of the current study sample, considering pertinent context-related factors and key external variables, and conducting cross-cultural comparative studies.

## Data Availability Statement

The raw data supporting the conclusions of this article will be made available by the authors, without undue reservation.

## Ethics Statement

The studies involving human participants were reviewed and approved by the Institutional Review Board of Guangzhou University. The patients/participants provided their written informed consent to participate in this study.

## Author Contributions

JQ and MW conceptualized the paper and MW contributed to the investigation and helped to perform the revision of the manuscript, and provided final approval for the manuscript, JQ contributed to the analysis of the data, and drafted the manuscript. All authors contributed to the article and approved the submitted version.

## Funding

This paper is supported by the National Natural Science Foundation of China (NSFC; 71972139; 71802119), the Soft Science Research Projects of Shanxi Province (2018041059–6), the Philosophy and Social Science Research Projects in Colleges and Universities of Shanxi Province (201803007).

## Conflict of Interest

The authors declare that the research was conducted in the absence of any commercial or financial relationships that could be construed as a potential conflict of interest.

## Publisher’s Note

All claims expressed in this article are solely those of the authors and do not necessarily represent those of their affiliated organizations, or those of the publisher, the editors and the reviewers. Any product that may be evaluated in this article, or claim that may be made by its manufacturer, is not guaranteed or endorsed by the publisher.

## Footnotes

^1^ https://www.wjx.cn

^2^WeChat is the most widely used social app installed on smart phones in China.

^3^¥5 yuan is about $ 0.8 dollars.
